# Importance of mosquito “quasispecies” in selecting an epidemic arthropod-borne virus

**DOI:** 10.1038/srep29564

**Published:** 2016-07-07

**Authors:** Marie Vazeille, Karima Zouache, Anubis Vega-Rúa, Jean-Michel Thiberge, Valérie Caro, André Yébakima, Laurence Mousson, Géraldine Piorkowski, Catherine Dauga, Marie-Christine Vaney, Mosè Manni, Giuliano Gasperi, Xavier de Lamballerie, Anna-Bella Failloux

**Affiliations:** 1 Department of Virology, Institut Pasteur, Arboviruses and Insect Vectors, 75724 Paris cedex 15, France; 2 Institut Pasteur of Guadeloupe, Laboratory of Medical Entomology, Environment and Health, Guadeloupe, BP 484, Morne Jolivière, 97183 Abymes, Guadeloupe; 3 Department of Infection and Epidemiology, Institut Pasteur, Environment and Infectious Risks/Laboratory for Urgent Response to Biological Threats (CIBU), Pole of Pathogen Genotyping, 75724 Paris cedex 15, France; 4 Centre de Démoustication/Conseil général de La Martinique, 97234 Fort-de-France, Martinique; 5 Aix Marseille Université, IRD French Institute of Research for Development, EHESP French School of Public Health, EPV UMR_D 190 ‘Emergence des Pathologies Virales’, 13005 Marseille cedex 05, France; 6 IHU Méditerranée Infection, APHM Public Hospitals of Marseille, 13385 Marseille cedex 05,France; 7 Center for Bioinformatics, BioStatistics and Integrative Biology, International Group for Data Analysis, 75724 Paris cedex 15, France; 8 Department of Virology, Institut Pasteur, Structural Virology, 75724 Paris cedex 15, France; 9 Department of Biology and Biotechnology “Lazzaro Spallanzani”, Laboratorio degli insetti di interesse agrario e sanitario,Università degli Studi di Pavia, Viale Ferrata 9, 27100, Pavia, Italy

## Abstract

Most arthropod-borne viruses (arboviruses), perpetuated by alternation between a vertebrate host and an insect vector, are likely to emerge through minor genetic changes enabling the virus to adapt to new hosts. In the past decade, chikungunya virus (CHIKV; *Alphavirus, Togaviridae*) has emerged on La Réunion Island following the selection of a unique substitution in the CHIKV E1 envelope glycoprotein (E1-A226V) of an East-Central-South African (ECSA) genotype conferring a higher transmission rate by the mosquito *Aedes albopictus*. Assumed to have occurred independently on at least four separate occasions, this evolutionary convergence was suspected to be responsible for CHIKV worldwide expansion. However, assumptions on CHIKV emergence were mainly based on viral genetic changes and the role of the mosquito population quasispecies remained unexplored. Here we show that the nature of the vector population is pivotal in selecting the epidemic CHIKV. We demonstrate using microsatellites mosquito genotyping that *Ae. albopictus* populations are genetically differentiated, contributing to explain their differential ability to select the E1-226V mutation. *Aedes albopictus*, newly introduced in Congo coinciding with the first CHIKV outbreak, was not able to select the substitution E1-A226V nor to preferentially transmit a CHIKV clone harboring the E1-226V as did *Ae. albopictus* from La Réunion.

Arboviruses have succeeded in escaping from their sylvatic cycles by changing their host range to increase infection rates in humans^1^,^2^,^3^,^4^. Subsequent epidemics may be facilitated by minor changes in the viral genome that enable the virus to adapt to new vectors, including invasive species. Competitive displacement of an indigenous mosquito species by an invasive competitor can have a significant impact on vector-borne diseases^5^,^6^. The mosquito species *Aedes albopictus* has attracted great interest as an invasive vector and potential competitor of resident mosquitoes, principally *Aedes aegypti*^5^,^6^,^7^,^8^. In the past decade, chikungunya virus (CHIKV; genus *Alphavirus*, family *Togaviridae*) has emerged in regions where *Ae. albopictus* has succeeded in occupying vacant ecological niches^6^.

CHIKV strains are distributed among three phylogroups: East/Central/South African (ECSA), West-African, and Asian. The CHIKV Indian Ocean lineage (IOL) emerged from the ECSA phylogroup and has spread throughout many tropical regions^9^. The IOL lineage was also introduced in Europe where autochthonous transmission was documented in Italy (2007)^10^ and in France (2010)^11^,^12^. It predominates among the currently circulating CHIKV strains in areas where the vector *Ae. albopictus* is present/dominant, in part due to selection of an *Ae. albopictus*-adaptive substitution in the CHIKV E1 envelope glycoprotein (E1-A226V). This substitution results in more efficient infection and dissemination in *Ae. albopictus*^13^,^14^. This region in E1 has been shown to play a role in viral entry *via* fusion of viral and endosomal membranes^15^. The adaptation of the ECSA genotype to *Ae. albopictus* is assumed to have occurred independently on at least four separate occasions^13^,^16^: on La Réunion Island (2004–2005)^4^ and India (2008)^17^ for the IOL lineage^18^, and in Cameroon (2006) and Gabon (2007) for the Central African lineage^19^.

In Africa, CHIKV was first isolated in Tanzania in 1952–1953^20^, the Democratic Republic of Congo (DRC) in 1958^21^ and again in 1960^22^. After almost 40 years of silence, two large CHIKV outbreaks were reported in Kinshasa, DRC in 1999 and 2000^23^ caused by ECSA CHIKV strains^24^. From 2004 to 2010, sequential epidemics were reported in Cameroon (2006) and Gabon (2007 and 2010) with CHIKV isolates harboring the E1-A226V substitution^16^ coinciding with the first detection of *Ae. albopictus* in the region^25^,^26^. In 2011, a CHIKV outbreak was reported in Congo with both *Ae. aegypti* and *Ae. albopictus* acting as vectors of CHIKV isolates presenting the *Ae. albopictus*-adaptive substitution E1-A226V^27^,^28^. Recent findings stressed the role of small genetic changes in the viral genome as the main factor leading to adaptation of new vectors for CHIKV emergence^13^,^14^,^29^. By contrast, thus far, there has been no evidence for a role of the genetic differentiation within mosquito populations (hereafter named quasipecies) on viral emergence.

Here, we performed 10 alternate passages mimicking host alternation between indigenous mosquitoes and a CHIKV presenting the original ECSA E1-226A isolated from Central Africa (DRC). We found that *Ae. albopictus* Congo behave differently from *Ae. albopictus* La Réunion; it was unable to (i) select the substitution E1-A226V and (ii) preferentially transmit CHIKV E1-226V when exposed to a blood-meal containing equal proportion of viral clones differing by a single mutation A or V at E1-226. Assuming that these differences can be attributed to mosquito genetics, we demonstrated that *Ae. albopictus* Congo were clearly different from *Ae. albopictus* La Réunion using mosquito genotyping with microsatellites, stressing the importance of the nature of the vector population in arbovirus emergence.

## Results

### *Ae. albopictus* transmit CHIKV Congo_2011 less efficiently than *Ae. aegypti*

**Figure 1:**
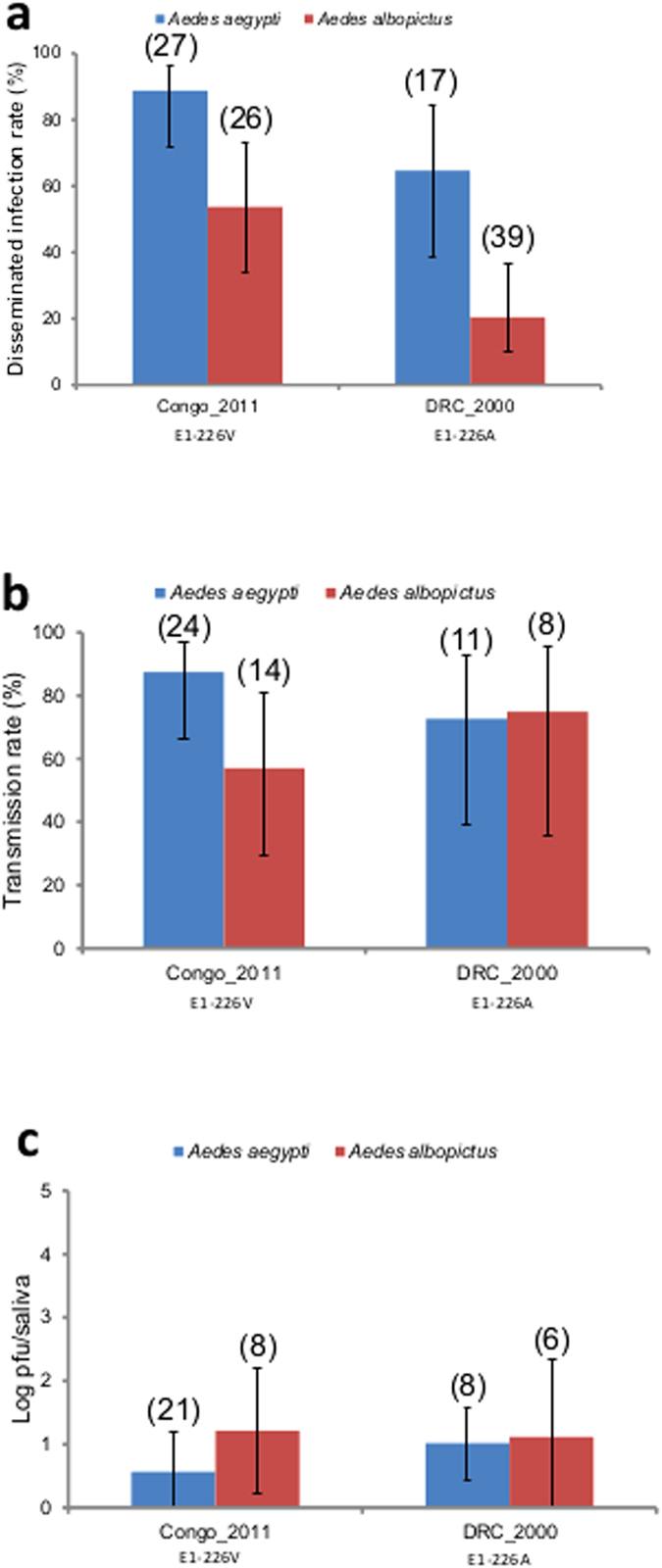
Disseminated infection rates, transmission rates and viral loads of saliva for mosquitoes infected with the two parental strains Congo_2011 and DRC_2000. (**a**) Disseminated infection rates, (**b**) transmission rates and (**c**) viral loads in saliva detected at 7 dpi for *Aedes aegypti* and *Aedes albopictus* from the Congo orally infected with the parental strains Congo_2011 and DRC_2000 provided at a titer of 10^6.5^ pfu/mL in the blood-meal. In brackets, the number of mosquitoes tested.

Both *Ae. aegypti* and *Ae. albopictus* from Congo were orally infected with DRC_2000 or Congo_2011 *via* an infectious blood-meal (Fig. 1). When analyzing viral dissemination at 7 days post-infection (dpi), disseminated infection rates (DIR) were higher in *Ae. aegypti* than in *Ae. albopictus* regardless of the viral strain (*P* < *0.05*, Fig. 1a). Transmission rates (TR), as determined by the presence of virus in saliva, were similar for both mosquito species infected with DRC_2000 and higher for *Ae. aegypti* infected with Congo_2011 (*P* < *0.05*, Fig. 1b). However, the viral titer present in saliva was higher in *Ae. albopictus* regardless of the viral genotype (*P* < *0.05*, Fig. 1c). Finally, in contrast to expectations, *Ae. albopictus* from Congo was not more efficient in transmitting CHIKV with the *Ae. albopictus*-adaptive mutation E1-226V.

### Alternate passages of CHIKV in human cells and mosquitoes do not impact viral dissemination and transmission in mosquitoes

**Figure 2:**
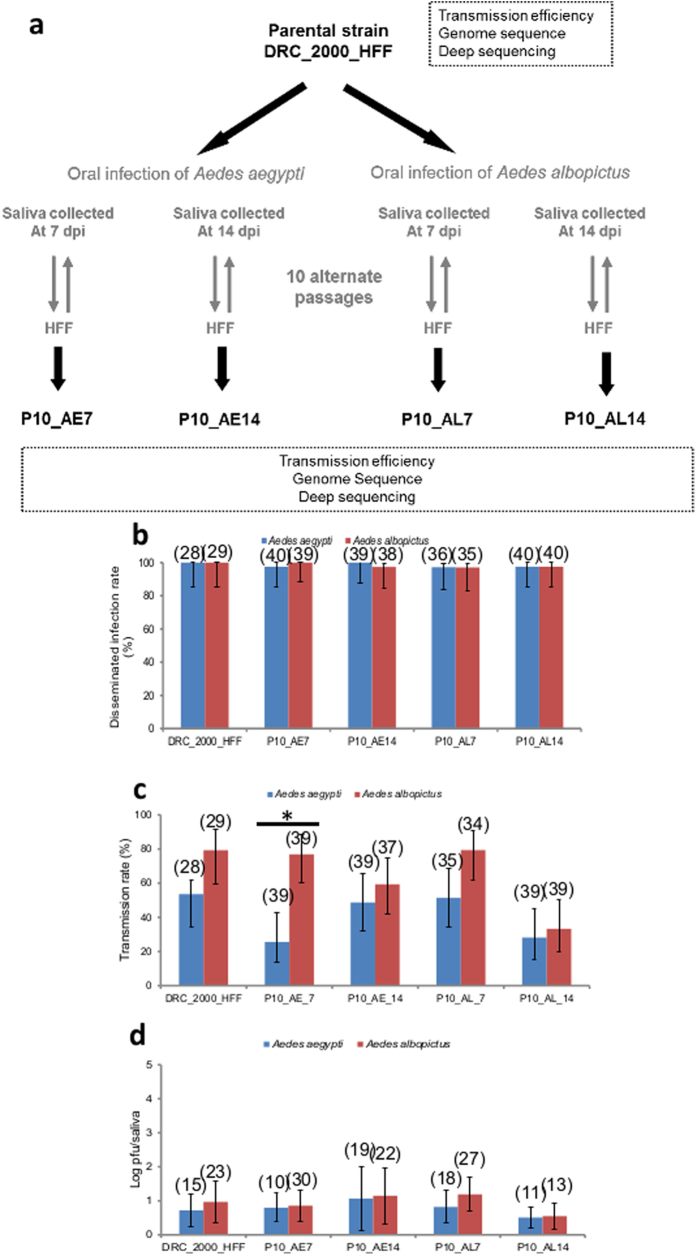
Experimental design for selecting CHIKV strains by alternate passages and vector competence analysis of samples. **(a)** The DRC_2000_HFF was alternatively passaged 10 times between mammalian HFF cells and mosquitoes (*Ae. aegypti* or *Ae. albopictus*) to mimic host alternation. Transmission efficiency was estimated after oral infection of mosquitoes. Genetic changes were identified by comparing the consensus sequence obtained with the reference CHIKV S27. Genetic diversity was determined by estimating variants in the viral population by deep sequencing. **(b)** Disseminated infection rates, **(c)** transmission rates and **(d)** viral loads of saliva at 7 dpi for mosquitoes orally infected with DRC_2000_HFF, and the 10 alternate passages between HFF cells and mosquitoes, provided at a titer of 10^6.5^ pfu/mL in the blood-meal. Error bars show the confidence intervals (95%). Significant p values are indicated by an asterix. In brackets, the number of mosquitoes tested.

Alternate passages between mosquitoes and human HFF cells were performed to experimentally attempt to select the mutation E1-226V from CHIKV DRC_2000 that has the E1-226A genotype. HFF cells were infected with CHIKV DRC_2000 followed by 10 alternate passages between mosquitoes and HFF cells and mosquitoes were analyzed for virus at 7 and 14 dpi (Fig. 2a). The resulting viral strains were orally provided to mosquitoes to determine their ability to disseminate and be transmitted. The first passage of CHIKV DRC_2000 on human HFF cells (DRC_2000_HFF) generated a viral strain causing an enhanced dissemination in both mosquito species ( < *0.05*, Fig. 2b), which did not however lead to a significant increase in transmission (*P* > *0.05*, Fig. 2c) or increased viral load in saliva (*P* > *0.05*, Fig. 2d). Overall, alternate passages of CHIKV did not improve the transmission potential of the virus when provided to the same mosquito species used for passages and in fact, reduced the viral titer in mosquito saliva in all cases except CHIKV P10_AE14.

### Substitutions are located in both non-structural and structural regions

To identify whether mutations generated by passages can be specific to one mosquito species, the viral genomes were sequenced. All viruses were grouped in an independent cluster within the ECSA phylogroup (Supplementary Fig. 1). When compared to the S27 strain isolated in 1952 in Tanzania^30^, the parental strain DRC_2000 and the viral population obtained after the 10^th^ alternate passage differed in 66 amino-acid substitutions: 40 in the non-structural proteins and 26 in the structural proteins. Of the 66 amino-acid substitutions, eight were found to be unique to one strain (Supplementary Table 1).

### Genetic diversity is increased in *Ae*. *aegypti* passaged strains

**Figure 3:**
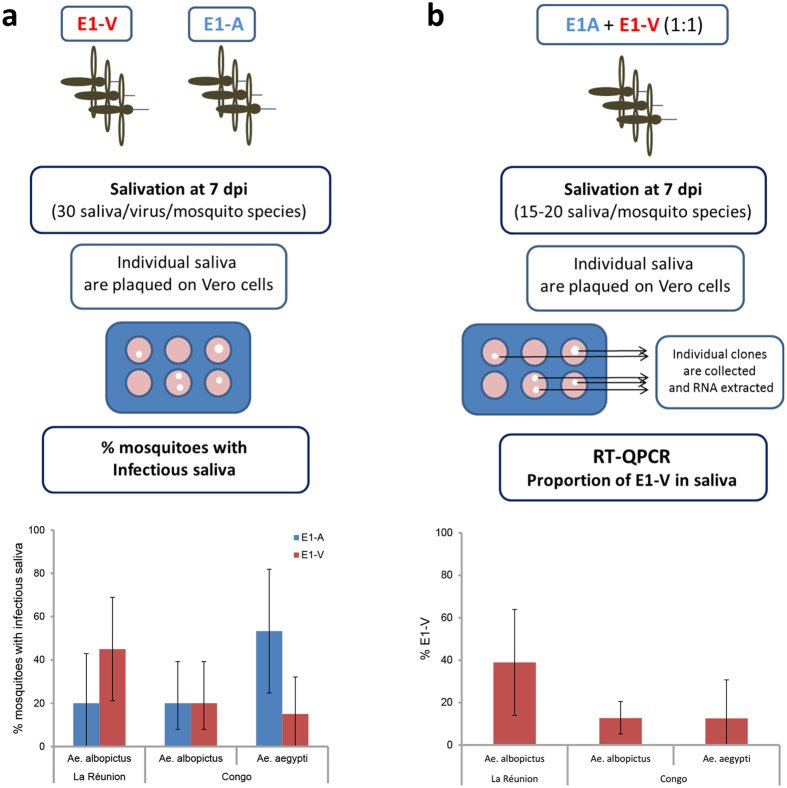
The biological clone E1-V IOL lineage is better transmitted by *Ae. albopictus* from La Réunion but not by *Ae. albopictus* from Congo. Both strains of *Ae. albopictus* (Congo and La Réunion) and *Ae. aegypti* from Congo were exposed to an infectious blood-meal containing an individual clone or both clones provided at equal titers. Saliva collected at 7 dpi were inoculated on Vero cells. (**a**) For mosquitoes infected with one viral clone, the percentage of infectious saliva was determined. (**b**) For mosquitoes infected with both viral clones, saliva were collected and inoculated on Vero cells. Then, lytic plaques were collected for RNA extraction and sequencing to define the identity of the amino-acid at the position E1-226. Proportion of E1-V among clones examined in saliva was estimated at 7 dpi for mosquitoes blood-fed with E1-A and E1-V provided at equal proportions. Error bars show the confidence intervals (95%).

Genetic diversity was examined by estimating mutation frequencies at each position of the viral genome by comparison to the DRC_2000_HFF reference sequence. Viral strains resulting from alternate passages harbored mainly fixed mutations with 13 detected in non-structural genes along with three mutations in the E2 gene (Table 1). The *Ae*. *aegypti* passaged strains: P10_AE7 and P10_AE14 contained a higher number of variants with fixed mutations than did *Ae*. *albopictus* passaged strains: P10_AL7 and P10_AL14 (Table 1). With the exception of the nsP2 A3210C (K510T) polymorphism, these fixed mutations segregated according to the mosquito species used for the alternate passages (Table 1). The three fixed mutations in the E2 glycoprotein detected in *Ae. aegypti* (I217V and G249R) and *Ae. albopictus* (G82R) were located on the surface of the virion without any obvious interaction with the position E1-226 (Supplementary Fig. 2). Unexpectedly, an amino-acid change C->Y at the position 483 of the nsP4 polymerase gene was identified in the CHIKV P10_AE7 strain (Table 1) coinciding with the substitution identified by^31^. All clones of the CHIKV P10_AE7 presented this mutation and none in the P10_AE14 strain (Extented Data Fig. 3a). The nsP4-C483Y substitution did not induce a replicative cost in either *Ae. aegypti* Aag2 or *Ae. albopictus* U4.4 cells (Extented Data Fig. 3b–e). The non-persistence of this mutation in passages initiated using late-collected saliva coincided with a higher viral load detected in saliva of mosquitoes infected with the P10_AE14 (Fig. 2d).

### The biological clone E1-V IOL is better transmitted by *Ae. albopictus* La Réunion but not by *Ae. albopictus* Congo

Competition assays were performed to assess transmission efficiencies of two biological clones E1-A (with E1-226A) or E1-V (with E1-226V) IOL in *Ae. albopictus* and *Ae. aegypti* from Congo in comparison with *Ae. albopictus* from La Réunion; saliva of 15–20 mosquitoes orally infected were examined at 7 dpi (Fig. 3). When provided alone (Fig. 3a), the E1-V biological clone presenting the *Ae. albopictus*-adaptive substitution E1-A226V was two-fold better transmitted by *Ae. albopictus* La Réunion as expected, whereas *Ae*. *albopictus* Congo did not exhibit any marked differences. On the other hand, *Ae. aegypti* Congo better transmitted the E1-A clone harboring the E1-226A substitution. In individuals exposed to a blood-meal containing an equal proportion of the two biological clones (Fig. 3b), the mean proportion of E1-V in saliva was 3 fold lower for *Ae. albopictus* Congo compared to *Ae. albopictus* La Réunion (*P* < *0.05*). The biological clone E1-V IOL was better transmitted by *Ae. albopictus* La Réunion but not by *Ae. albopictus* Congo.

### *Ae. albopictus* Congo and *Ae. albopictus* La Réunion are genetically distinct

**Figure 4:**
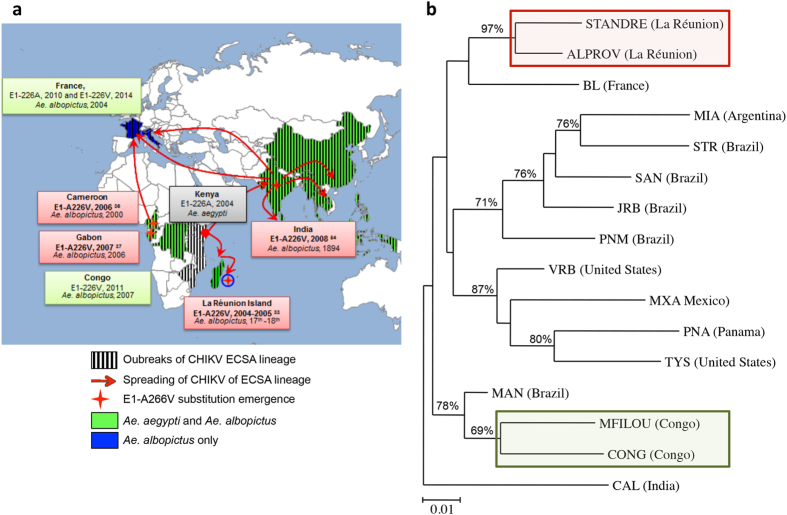
Genetic differentiation of *Aedes albopictus* populations. **(a**) Map showing the four E1-A226V substitution emergence events in La Réunion Island^33^, India^55^, Cameroon^36^ and Gabon^37^ and spreading of CHIKV ECSA lineage correlated with the presence of *Ae. albopictus*. Date of introduction or first description of *Ae. albopictus* in the country is indicated. **(b)** Neighbor-joining cluster analysis (unrooted) based on Cavalli-Sforza and Edwards’s chord distance. Apparent root (CAL from India) is for visual purposes only. Numbers indicate bootstrap values above 65%. Genotyping included DNA extraction from 30 mosquitoes (15 males and 15 females), PCR amplification of 11 microsatellites and sequencing of fragments for scoring haplotypes. A dendrogram based on microsatellite Cavalli-Sforza & Edwards’s genetic distance clustering by the NJ method was constructed using 16 *Ae. albopictus* populations: ALPROV, Saint-Denis, La Réunion; BL, Bar-sur-Loup, France; CAL, Calcutta, India; CONG, Brazzaville, Congo; JRB, Jurujuba, Brazil; MAN, Manaus, Brazil; MFILOU, Brazzaville, Congo; MIA, Misiones, Argentina; MXA, Tapachula, Mexico; PNA, Colon, Panama; PNM, Parnamirim, Brazil; SAN, Santos, Brazil; STANDRE, Saint-André, La Réunion; STR, Santarém, Brazil; TYS, Tyson, United States; VRB, Florida, United states. F1 mosquitoes were used except for the lab colony CAL. The map was modified using PowerPoint from http://www.powerpointslides.net/powerpointgraphics/powerpointmaps.html using a map previously published in^56^.

The location of the four E1-A226V substitution emergence events are shown on the map (Fig. 4a): La Réunion Island (2004–2005)^4^ and India (2008)^17^ for the IOL lineage^18^, and Cameroon (2006) and Gabon (2007) for the Central African lineage^19^. Date of introduction or first description of *Ae. albopictus* is indicated for each concerned country. Genetic relatedness between 16 *Ae. albopictus* populations was assessed by genotyping mosquitoes using 11 microsatellites. All loci were polymorphic with 4–21 alleles scored (Supplementary Table 2). The neighbor-joining tree based on Cavalli-Sforza and Edwards’s chord distances showed three distinct groups with high bootstrap support at nodes separating them (Fig. 4b). The two most distant groups included the two populations from La Réunion (STANDRE and ALPROV) and the two populations from Congo (MFILOU and CONG). Mosquitoes from La Réunion were grouped with *Ae. albopictus* from France (BL), whereas mosquitoes from Congo were grouped with *Ae. albopictus* from India (CAL) suggesting different sources of introduction. A third group comprising nine populations from America was subdivided into two subgroups: a subgroup associating mosquitoes from South America and another subgroup comprised of mosquitoes from Central/North America.

## Discussion

A key feature of many invasive species is their ability to displace and eventually replace the indigenous species that occupies the same ecological niche. Once it arrives in a region, *Ae. albopictus* tends to displace the indigenous *Ae. aegypti* through its larval competitive advantage for resources^25^,^32^,^33^ and which may alter vector competence^34^,^35^. Cameroon^36^, Gabon^37^, or the Central African Republic^38^ were the main source of the *Ae. albopictus* invasion into Congo. Based on microsatellite genotyping, we showed that *Ae. albopictus* from Congo are genetically different from *Ae. albopictus* from La Réunion where the *Ae. albopictus*-adaptive substitution E1-A226V has been selected. Furthermore, when exposed to an equal proportion of CHIKV clones differing by a single substitution A or V at E1-226, *Ae. albopictus* from Congo did not preferentially transmit the clone harboring the E1-226V mutation as expected.

CHIKV has recently spread throughout many tropical regions due in part to an amino-acid substitution in the E1 glycoprotein (E1-A226V) that enhances infectivity for *Ae. albopictus*. It is assumed that this mutation has been selected for on at least four separate occasions including Central Africa, and appears to coincide with the occurrence of *Ae. albopictus* as a CHIKV vector^13^,^16^. Cases of CHIK disease were first reported in Brazzaville (Congo) in June 2011. This emergence was assumed to be related to a host switching event from *Ae. aegypti* to the Asian *Ae. albopictus* as both mosquito species were found naturally infected in the field^28^ in a situation similar to that observed in Cameroon and Gabon^26^,^39^. We compared the native *Ae. aegypti* and the recently introduced *Ae. albopictus* collected from the same breeding site and showed that the Congo *Ae. albopictus* was less able to transmit the most recent epidemic CHIKV strain, Congo_2011, which harbours the E1-226V mutation. This was surprising, as it has been shown that under some circumstances, this mutation enhances CHIKV transmission by *Ae. albopictus*^13^,^14^.

The CHIKV strain Congo_2011 differs mainly from DRC_2000 at two amino-acid positions. CHIKV DRC_2000 possesses the prototypic E1-226A and E2-337V residues, while CHIKV Congo_2011 contains the E1-226V and E2-377I mutations (Supplementary Table 3). We attempted to experimentally enhance the potential of the DRC_2000 strain to be transmitted by *Ae. albopictus* by alternately passaging the virus between either *Ae. aegypti* or *Ae. albopictus* mosquitoes from Congo and human cells. After the 10^th^ passage, we conducted genome sequence analysis and showed that genetic changes in the consensus sequence were located in both non-structural and structural regions (Table 1). We then analyzed the genetic diversity of these viral populations using deep sequencing and showed that the proportion of fixed mutations (frequency >80%) was lower in sequences derived from virus passaged in *Ae. albopictus*. These findings suggest that the environment within *Ae. albopictus* exerts a stronger selective pressure on the virus and acts to bottleneck the viral genetic diversity with consequences on CHIKV adaptive evolution^40^.

Under the selective pressure of *Ae. aegypti* however, the frequency of mutations within the viral population was higher. In addition, we observed a higher number of mutations in virus collected at 14 dpi when compared to 7 dpi suggesting that there may be a temporal effect on population diversity. Of particular interest, we isolated a fixed mutation in CHIKV P10_AE7, which results in an amino-acid substitution (nsP4-C483Y), previously described by^31^. Located within the viral RNA dependent RNA polymerase (RdRp), this mutation was shown to cause an increase in replication fidelity. This results in the generation of a viral population with reduced genetic diversity, potentially leading to reduced viral fitness both in *Ae. aegypti* mosquitoes and in vertebrate hosts^31^. Unsurprisingly, this mutation was not recovered from any other sample populations further supporting this hypothesis. Nevertheless, the CHIKV nsp4 C483Y mutation did not appear to impact viral replication as P10_AE7 replicates well in both *Ae. aegypti* Aag2 and *Ae. albopictus* U4.4 cells (Supplementary Fig. 3b–e). In *Ae. aegypti* mosquitoes, virus recovered from passages using late-collected saliva at 14 dpi showed both increased genetic diversity (Table 1) and increased viral load in saliva (Fig. 2d). These data suggest that genetic diversity of CHIKV is important in maximizing viral transmission. Two amino-acid substitutions in the E2 glycoprotein at positions 217 and 249 (Table 1) were also identified. Neither of these residues have been shown to interact with E1 position 226 and in fact, are located on the outside of the trimeric spike where they could potentially play a role in the induction of the host protective immune response^41^ or interaction of the virion with host receptor proteins^42^ (Supplementary Fig. 2).

*Ae. albopictus* from Congo are different from those on La Réunion when comparing their transmission potential for CHIKV IOL. When exposed to an equal proportion of viral clones differing by a single substitution A or V at E1-226, *Ae. albopictus* from Congo did not preferentially transmit the clone harboring the *Ae. albopictus*-adaptive substitution E1-A226V as expected (Fig. 3b). One explanation for this is that *Ae. albopictus* from Congo are genetically different from *Ae. albopictus* from La Réunion; geographically-distant populations have lower genetic connectedness and higher population genetic differentiation with potential consequences for their transmission of infectious agents. We showed using mosquito genotyping with microsatellites that *Ae. albopictus* from Congo and from La Réunion formed divergent groups (Fig. 4b) with probably a different history of colonisation^33^,^43^. The process of ongoing competitive displacements of *Ae. aegypti* by *Ae. albopictus* in Central Africa^44^ provides a situation different from La Réunion Island where *Ae. albopictus* was the unique vector^32^. The selection of the mutation E1-226V of an ECSA genotype conferring a higher transmission by *Ae. albopictus* was unlikely to have been the scenario that occurred in the Congo as it was in La Réunion. Therefore, CHIKV ECSA E1-226V was most likely introduced into the Congo rather than selected *in situ*. Thus, attention should be paid to the existence of specific interactions between mosquito and virus genotypes that have differing potential for leading to an emergence event.

## Methods

### Ethics Statement

The Institut Pasteur animal facility has received accreditation from the French Ministry of Agriculture to perform experiments on live animals in compliance with the French and European regulations on care and protection of laboratory animals. This study was approved by the Institutional Animal Care and Use Committee (IACUC) at the Institut Pasteur. No specific permits were required for the described field studies in locations, which are not protected in any way and did not involve endangered or protected species.

### Mosquitoes

Eggs were collected from ovitraps placed in the city of Brazzaville (Congo) on the ORSTOM campus in august 2011 and sent to the Institut Pasteur in Paris where they were reared in standardized conditions. After morphological identification, *Ae. aegypti* and *Ae. albopictus* adults were placed in different cages and reared at 28 ± 1°C, at relative humidity of 80% and a light:dark cycle of 16 h:8 h. A constant supply of 10% sucrose was provided to adults. To produce eggs, females were fed three times a week on anesthetized mice (OF1 mice from Charles River laboratories, France). Oral infection experiments were performed using mosquitoes from the F2-F4 generations. The F12 generation of *Ae. albopictus* Providence (ALPROV) collected in 2007 on La Réunion Island was used for comparison.

### Cell cultures

C6/36 (*Ae. albopictus*) cells were maintained at 28 °C in Leibovitz L-15 medium supplemented with non-essential amino-acids (1X), 10% fetal bovine serum (FBS), 100 units/mL penicillin and 100 μg/mL streptomycin. They were used for production of parental viral stocks. Vero (green monkey kidney) cells used for CHIKV titrations were maintained at 37 °C, 5% CO_2_ in Dulbecco’s Modified Eagle medium (DMEM) with 10% FBS, 100 units/mL penicillin and 100 μg/mL streptomycin. HFF (Human Foreskin Fibroblast) cells were maintained at 37 °C, 5% CO_2_ in Dulbecco’s Modified Eagle medium (DMEM) supplemented with pyruvate, 10% FBS, 100 units/mL penicillin and 100 μg/mL streptomycin. These cells were used for the alternate passages as a proxy to mimic the human host. Two other types of mosquito cells were used to assess replication kinetics: (i) an *Ae. aegypti*-derived Aag2 cells and (ii) an *Ae. albopictus*-derived U4.4 cells. Aag-2 cells were maintained in Schneider Drosophila medium, 10% FBS and 1% P/S and U4.4 cells were maintained in L-15 medium supplemented with 10% FBS and 10% tryptose phosphate broth at 28 °C.

### Viruses

The CHIKV DRC_2000 and Congo_2011 strains were isolated from patients during the epidemics of 2000 in the DRC and 2011 in Congo and were both provided by the French National Reference Center for Arboviruses at the Institut Pasteur. Both isolates belong to the ECSA phylogroup and the Central African lineage. The DRC_2000 and Congo_2011 differ in two major substitutions in E1-226 and E2-337: the DRC_2000 strain harbours E1-226A and E2-337V whereas the Congo_2011 strain presents E1-226V and E2-337I (Supplementary Table 3). Viral stocks were produced on C6/36 and viral titers were determined *via* plaque assay as previously described^14^. In addition, biological clones E1-A and E1-V of IOL lineage which differ by a single position E1-226 from an alanine (A) to a valine (V), produced by plaque purification from two CHIKV isolates from La Réunion Island, respectively CHIKV 06.21 and CHIKV 05.115 were also used^14^,^45^.

### Mosquito oral infections

Infection assays were performed with one-week-old females starved 24 hrs prior to infection in a BSL-3 laboratory. Mosquitoes were allowed to feed for 15 min through a piece of pork intestine (for *Ae. aegypti*) or chicken skin (for *Ae. albopictus*) covering the base of a Hemotek feeder containing the infectious blood-meal maintained at 37 °C. The blood-meal was composed of 1/3 of viral supernatant, 2/3 of washed rabbit erythrocytes isolated from arterial blood, and adenosine triphosphate at a final concentration of 10^−3^ M^46^. To assess the vector competence, adult females of F2 generation (for CHIKV DRC_2000 or Congo_2011 strains) and of F3/F4 generation (for the four CHIKV strains resulting from the 10^th^ passage; Fig. 1) were exposed to infectious blood-meals at a titre of 10^6.5^ pfu (plaque forming units)/mL and engorged females were separated and incubated under controlled conditions (28 ± 1°C, relative humidity of 80%, light:dark cycle of 16 h:8 h). At 7 dpi, vector competence was assessed based on two phenotypes: (i) viral dissemination from the midgut into mosquito general cavity and (ii) transmission potential with virus detected in mosquito saliva. Disseminated infection rate (DIR) was defined as the percentage of engorged mosquitoes with virus detected in heads suggestive of successful viral dissemination from the midgut. Transmission rate (TR) was calculated as the percentage of mosquitoes with viral particles detected in saliva among mosquitoes that developed a disseminated infection. Saliva was collected as described in Dubrulle *et al*.^47^. Briefly, wings and legs were removed from each individual and its proboscis was inserted into a 20 μL tip containing 5 μL of FBS. After 45 min, FBS containing saliva was expelled in 45 μL of Leibovitz L15 medium (Invitrogen) for titration. Experimentally induced salivation is widely used to demonstrate the transmission of pathogens ingested by hematophagous insects^48^. DIR and TR were calculated by titrating head homogenates or saliva on Vero cells as previously described^14^.

### Competitive assays between two viral clones E1-V and E1-A from IOL lineage

Competition assays were performed by providing the two biological clones E1-A (with E1-226A) and E1-V (with E1-226V) from IOL lineage in an equal proportion (1:1) to mosquitoes in blood-meals prepared at a final titre of 10^6.5^ pfu/mL. At 7 dpi, viruses were isolated from saliva. Briefly, viruses were used to infect six-well-plates containing confluent monolayers of Vero cells. Cells were incubated for 3 days at 37 °C and 5% CO_2_ under an overlay consisting of DMEM (1X) with 2% FBS, 1% L-Glutamine, 1% agarose and 1% penicillin/streptomycin/amphotericin (Invitrogen). For each mosquito species, 30 saliva samples were analyzed and lytic plaques were removed by suction using a pipette. Each agarose plug containing an individual clone was dissolved overnight at + 4°C in 50 μL of DMEM. RNA was extracted using NucleoSpin RNA kit (Macherey Nagel) according to the manufacturer’s instruction. A one-step RT-PCR reaction targeting a region comprising the position 226 in the E1 gene was performed using the Titan One Tube kit (Roche). The sequencing reaction was conducted using the ABI Prism BigDye Terminator Cycle Sequencing Ready Reaction kit version 3.1 (Applied Biosystems)^45^.

### Alternate passages of the parental CHIKV DRC_2000 strain

Alternate passages are summarised on Fig. 2. Before alternate passages, a stock of DRC_2000 was produced on HFF human cells (DRC_2000_HFF) using the DRC_2000 parental strain at a multiplicity of infection (MOI) of 0.1. For the first passage in mosquitoes, the F2 generation of both species (*Ae. aegypti* and *Ae. albopictus*) were orally infected with the produced DRC_2000_HFF supernatant provided in a blood-meal at a final titer of 10^6.5^ pfu/mL. Engorged mosquitoes were incubated at 28 °C for either 7 or 14 days and then processed for saliva collection. Saliva was pooled by species and day of collection (4 samples, 20 salivations per sample) and the volume of each sample adjusted to 600 μL with DMEM prior to filtration through a Millipore H membrane (0.22 μm). An aliquot of 300 μL of each sample was used to inoculate a sub-confluent flask (25 cm^2^) of HFF cells (considered as the first passage of saliva on human cells). After 1 hour, the inoculum was discarded and cells rinsed once with DMEM medium. 5 mL of DMEM medium complemented with 2% FBS were added and cells were incubated for 3 days at 37 °C. Cell culture supernatants were then collected and stored at −80 °C until used. For each passage and each condition (P10_AE7, P10_AE14, P10_AL7 and P10_AL14, where AE and AL stand for *Ae. aegypti* and *Ae. albopictus*, respectively) (Fig. 2a), saliva were pooled from 10 to 20 individuals depending on the mosquito feeding rate. Passages P2 to P6 were performed with mosquitoes of the F3 generation and passages P7 to P10 with mosquitoes of the F4 generation. HFF supernatants collected at each passage were used undiluted for the next mosquito blood-meal without titration.

### Replication kinetics

To measure viral replicative fitness, growth curves were conducted in *Ae. aegypti* Aag2 and *Ae. albopictus* U4.4 cells. Confluent cell monolayers were prepared and inoculated with viruses simultaneously in duplicates at a MOI of 0.1 pfu/cell. Cells were incubated for 1 hour in appropriate conditions. Viral inoculum was removed and cell monolayers were washed 3 times with PBS to eliminate unbound virus. Two mL of medium supplemented with 5% FBS were then added and cells were incubated at 28 °C. At various times (0, 4, 6, 8, 10 and 24 hrs) post-inoculation, supernatants were collected and titrated by Vero plaque assays. To estimate the number of RNA copies, RNA was extracted from the same samples using Nucleospin RNA II kit (Macherey-Nagel) and a one-step qRT-PCR was performed to detect the number of viral RNA copies according to protocols described by Vazeille *et al*.^14^. Primers have been designed in the E2 structural gene: sense Chik⁄E2⁄9018⁄+ (CACCGCCGCAACTACCG) and anti-sense Chik⁄E2⁄9235⁄− (GATTGGTGACCGCGGCA).

### Genome sequencing

Viral RNA was extracted from supernatants of DRC_2000, P10_AE7, P10_AE14, P10_AL7 and P10_AL14. RT-PCR was performed using SuperScript One-Step RT-PCR with platinum Taq (Invitrogen) using 21 sets of primers targeting the complete CHIKV genome as described by Schuffenecker *et al*.^4^. Amplicon sequencing reactions were performed using Big Dye Terminator v1.1 cycle sequencing kit (Applied Biosystems) and sequencing was performed using an ABI3730XL sequence analyzer (Applied Biosystems). Sequence analysis, contig assembly and sequence alignments were performed using BioNumerics version 6.5 (Applied-Maths, Saint-Martens-Latem, Belgium). For phylogenetic analysis, maximum-likelihood tree was constructed using MEGA version 5 (www.megasoftware.net), based on the Tamura-Nei model. Reliability of nodes was assessed by bootstrap resampling with 1,000 replicates.

### Viral diversity

Whole genome sequences (excluding the first 19 nucleotides of the 5′UTR and the 41 nucleotides upstream the polyA tail) were determined for DRC_2000_HFF and the last passages (P10_AE7, P10_AE14, P10_AL7 and P10_AL14) using the Ion PGM Sequencer (Life Technologies) as described by Rothberg *et al*.^49^ and sequence analysis was conducted using CLC Genomics Workbench 6 software. For deep sequencing, a set of four primer pairs (Supplementary Table 4) was used to generate amplicons with 3 μL of nucleic acid extract and the Superscript III One-Step RT-PCR Platinum TaqHifi kit (Life Technologies) according to manufacturer’s instructions using the following cycling parameters: 50 °C for 30 min, 94 °C for 2 min followed by 45 cycles of 94 °C for 15 sec, 56 °C for 30 sec and 68 °C for 4 min. PCR products were verified by gel electrophoresis and amplicons were purified using Amicon Ultra – 0.5 mL 30 K kit (Millipore) according to the manufacturer’s instructions. For each sample, an equimolar mix of all amplicons was used to build a library and produce the corresponding sequences for the Ion PGM Sequencer according to the manufacturer’s instructions. The reads obtained were trimmed: first using quality score and then by removing the primers used for amplification. Reads were mapped to the genome sequence of CHIKV DRC_2000_HFF, which was used as a reference. Mutation frequencies (proportion of viral genomes with the mutation) at each position were calculated as the number of reads with a mutation compared to the reference divided by the total number of reads at that site. Only substitutions with a mutation frequency ≥5% were considered significant for further analysis.

### Mosquito genotyping

DNA was extracted from each mosquito with a commercial purification kit (Nucleospin 96 DNA Tissue kit, Macherey-Nagel) following manufacturer’s instructions for vacuum processing. Briefly, 30 mosquitoes (15 males and 15 females) per population were individually grounded in 180 μL lysis buffer supplemented with 25 μL of Proteinase K. The homogenates were then passed through columns allowing binding of total nucleic acids. Silica membranes were further desalted and DNA was finally eluted in 100 μL of elution buffer. Eleven microsatellites were amplified by PCR using specific primers flanking the repeated region^50^. PCR reaction mixtures consisted of 50 ng genomic DNA, 1x PCR buffer, 1.5 mM MgCl2, 0.27 mM dNTPs (Invitrogen), 1 U *Taq* polymerase (Invitrogen), and 10 μM of each primer, one of which was 5′ labelled with a fluorescent dye, in a final volume of 15 μL. PCR cycling conditions were 94 °C for 5 min, 30 cycles of 94 °C for 30 s, 59 °C for 30 s, and 72 °C for 30 s, followed by a final extension step of 72 °C for 7 min. Aliquots of PCR products were separated by electrophoresis on 2% agarose gels stained with ethidium bromide, and visualized under UV light. Each PCR product was then diluted 1:10 in ddH2O water and 2 μL of this dilution was added to 10 μL of a mixture of deionized formamide and GeneScan-500 ROX size standard (Applied Biosystems). The fragments were then resolved on an ABI3730XL sequence analyzer (Applied Biosystems). Data were analysed using GeneScan and Genemapper software. The program TANDEM^51^ was used to limit ambiguous genotyping.

### Statistical/Phylogenetical analysis

Statistical analyses were conducted using the STATA software (StataCorp LP, Texas, and USA). Rates/proportions (disseminated infection rate, transmission rate, percentage of infectious saliva, proportion of E1-V clones) were compared using Chi square test. The numbers of infectious particles in saliva were compared using Kruskal-Wallis test. P-values > 0.05 were considered non-significant. Allelic frequencies were obtained using the GENEPOP software (version 4.0) of Raymond & Rousset^52^. To assess relatedness among populations, Cavalli-Sforza & Edwards’s^53^ chord distance for each pair of populations was calculated in PHYLIP 3.69 (GENDIST module) and the resulting distance matrix was used to create a dendrogram. A neighbor-joining tree was constructed with node confidence inferred via 100 bootstrap replicates in PHYLIP 3.69 (modules SEQ- BOOT, GENDIST, NEIGHBOUR and CONSENSE). The neighbor-joining network should not be taken as a true phylogeny since microsatellites are not ideal markers for recovering evolutionary history^54^. Rather, the analysis should be considered as a method to visualize discrete genetic clustering of populations.

## Additional Information

**How to cite this article**: Vazeille, M. *et al*. Importance of mosquito “quasispecies” in selecting an epidemic arthropod-borne virus. *Sci. Rep.* **6**, 29564; doi: 10.1038/srep29564 (2016).

## Supplementary Material

Supplementary Info File #1

## Figures and Tables

**Table 1:** Population genetic diversity in the four selected CHIKV strains.

nt position	nt change	Gene	aa change	Frequency of mutations (%)			
				*Ae. aegypti*		*Ae. albopictus*	
				P10_AE7	P10_AE14	P10_AL7	P10_AL14
562	A -> G	nsp1	162 (L)^#^		99.9		
978	C -> T	nsp1	T301I		99.8		
1017	C -> T	nsp1	T314M		100		
1851	G -> A	nsp2	A57T	99.9			
2635	C -> T	nsp2	318 (T)^#^	99.8			
3210	A -> C	nsp2	K510T	99.6	99.3	99.7	
3734	A -> G	nsp2	I685D				99.9
4424	G -> A	nsp3	G117R				99.9
4897	C -> T	nsp3	274 (T)^#^		99.8		
5942	C -> T	nsp4	P93S	97.2		93	
6157	C -> T	nsp4	164 (F)^#^		98.7		
6724	C -> T	nsp4	353 (F)^#^				100
7113	G -> A	nsp4	C483Y	99.9			
8785	G -> A	E2	G82R			12.2*	
9190	A -> G	E2	I217V		99.9		
9286	G -> A	E2	G249R		98.8		

nt, nucleotide; aa, amino-acid; ^#^no amino-acid change, synonymous mutation; *minority variant not found in the consensus sequence; P10_AE7, 10^th^ passage between HFF cells and *Ae. aegypti* initiated with saliva collected at 7 dpi; P10_AE14, 10^th^ passage between HFF cells and *Ae. aegypti* initiated with saliva collected at 14 dpi; P10_AL7, 10^th^ passage between HFF cells and with *Ae. albopictus* initiated with saliva collected at 7 dpi; P10_AL14, 10^th^ passage between HFF cells and *Ae. albopictus* initiated with saliva collected at 14 dpi. The DRC_2000_HFF was used as the reference genome.
