# Supplementary material for: Importance of mosquito “quasispecies” in selecting an epidemic arthropod-borne virus
Source: Sci Rep. 2016 Jul 7;6:29564. doi: 10.1038/srep29564 (PMC4935986; doi:10.1038/srep29564)
Supplement: Supplementary Info File #1 [file srep29564-s1.doc]

**Importance of mosquito quasispecies in selecting an epidemic arthropod-borne virus.**

Marie Vazeille^1^, Karima Zouache^1^, Anubis Vega-Rúa^1,2^ Jean-Michel Thiberge^3^, Valérie Caro^3^, André Yébakima^4^, Laurence Mousson^1^, Géraldine Piorkowski^5,6^, Catherine Dauga^7^, Marie-Christine Vaney^8^, Mosè Manni^9^, Giuliano Gasperi^9^, Xavier de Lamballerie^5,6^, and Anna-Bella Failloux^1,*^

**Supplementary Figure 1 | Phylogenetic tree derived from complete CHIKV sequences available in the GenBank library**. Phylogeny reconstruction was achieved using the ML technique with 1,000 replicate bootstrap values (MEGA version 5.2.1.). Bootstrap support values are indicated at major nodes. Scale bar indicates the number of base substitutions per site. Our strains are indicated in blue.


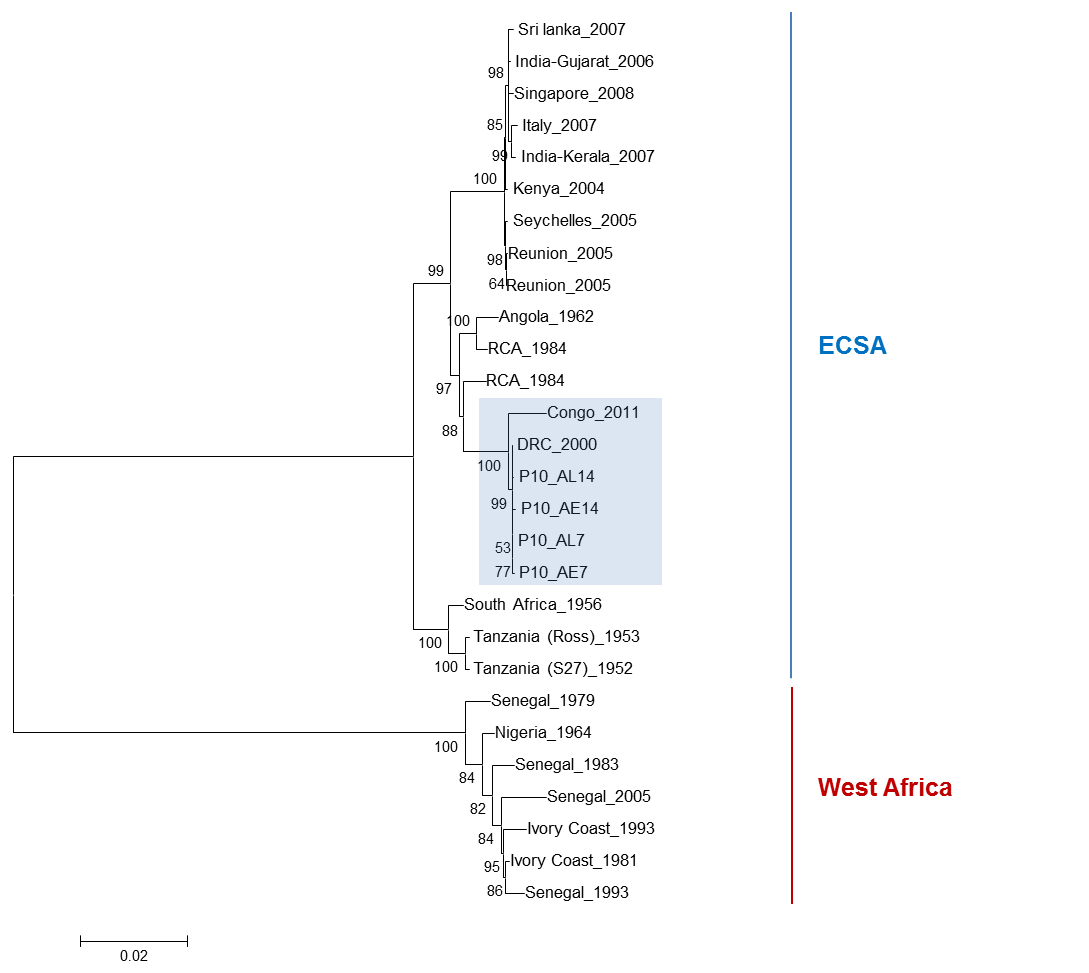


**Importance of mosquito quasispecies in selecting an epidemic arthropod-borne virus.**

Marie Vazeille^1^, Karima Zouache^1^, Anubis Vega-Rúa^1,2^ Jean-Michel Thiberge^3^, Valérie Caro^3^, André Yébakima^4^, Laurence Mousson^1^, Géraldine Piorkowski^5,6^, Catherine Dauga^7^, Marie-Christine Vaney^8^, Mosè Manni^9^, Giuliano Gasperi^9^, Xavier de Lamballerie^5,6^, and Anna-Bella Failloux^1,*^

**Supplementary Figure 2 | Mapping of the mutations on the surface of the Chikungunya virus. a,** Surface of the virus. The envelope proteins E2 and E1 are displayed in red and grey, respectively. The E1-226 position detected in the Indian Ocean isolates is shown as a black sphere. The mutations E2-G249R, E2-G82R and E2-I217V are localized on the surface with spheres colored respectively in green, yellow and cyan. The spike containing the trimer of E2E1 is outlined by a black triangle. **b**, Zoom view of the spike defined by the black triangle in **a**. The proteins E1, E2 and the mutations are color-coded as in **a**. This figure was prepared using PyMOL (The PyMOL Molecular Graphics System, Version 1.5.0.4 Schrödinger, LLC).


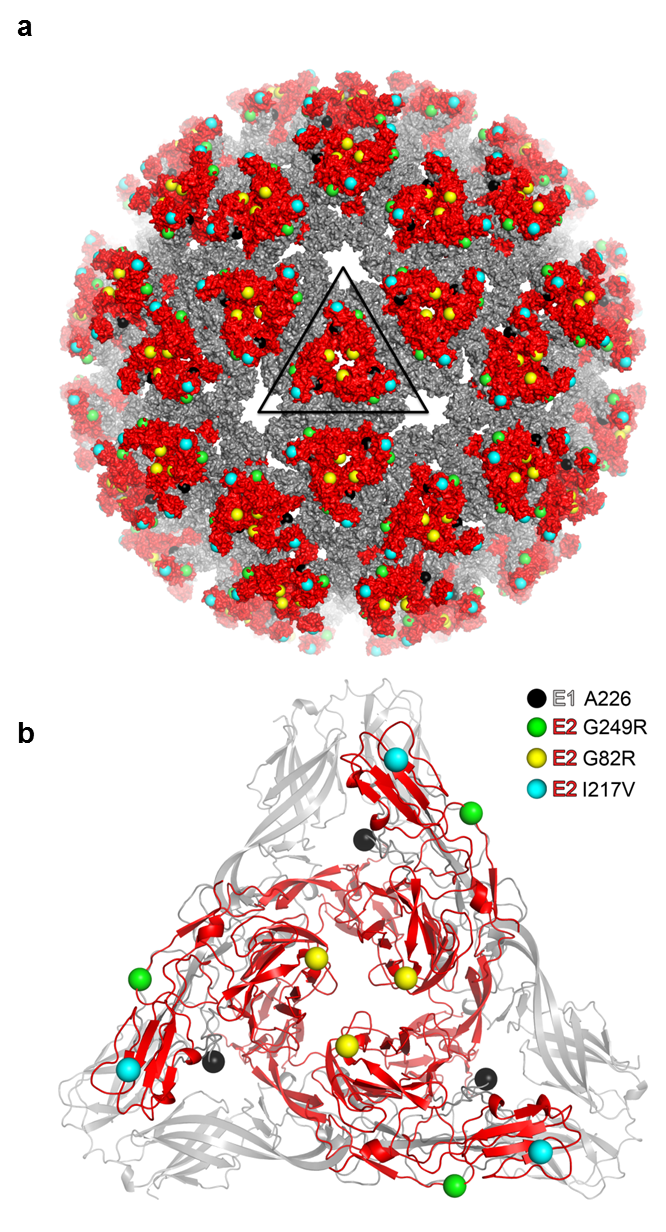


**Importance of mosquito quasispecies in selecting an epidemic arthropod-borne virus.**

Marie Vazeille^1^, Karima Zouache^1^, Anubis Vega-Rúa^1,2^ Jean-Michel Thiberge^3^, Valérie Caro^3^, André Yébakima^4^, Laurence Mousson^1^, Géraldine Piorkowski^5,6^, Catherine Dauga^7^, Marie-Christine Vaney^8^, Mosè Manni^9^, Giuliano Gasperi^9^, Xavier de Lamballerie^5,6^, and Anna-Bella Failloux^1,*^

**Supplementary Figure 3 | Viral replication of the CHIKV P10_AE7 passage containing the substitution nsp4-C483Y increasing replication fidelity. a,** a fragment of the nsP4 containing the position 483 was amplified and sequenced from 20 biological clones isolated from the supernatant of P10_AE7 and P10_AE14 strains to estimate the proportion of infectious particles harboring this mutation nsp4-C483Y; primers were CHIKV6732F 5’-TCATAGCCGCACACTTTAAGC-3’ and CHIKV7495R 5’-AGGACCGCCGTACAAAGTTAC-3’. **b, c,** Aag2 and U4.4 cells were inoculated in duplicate with CHIKV P10_AE7 (gray) and DRC_2000 (black) at 0.1 pfu/cell and titers of supernatants were determined by Vero plaque assay at different hours post-infection. **c**, **d**, genome copy were also measured by quantitative RT-PCR; mean values ± SD are shown at different hours post-infection.


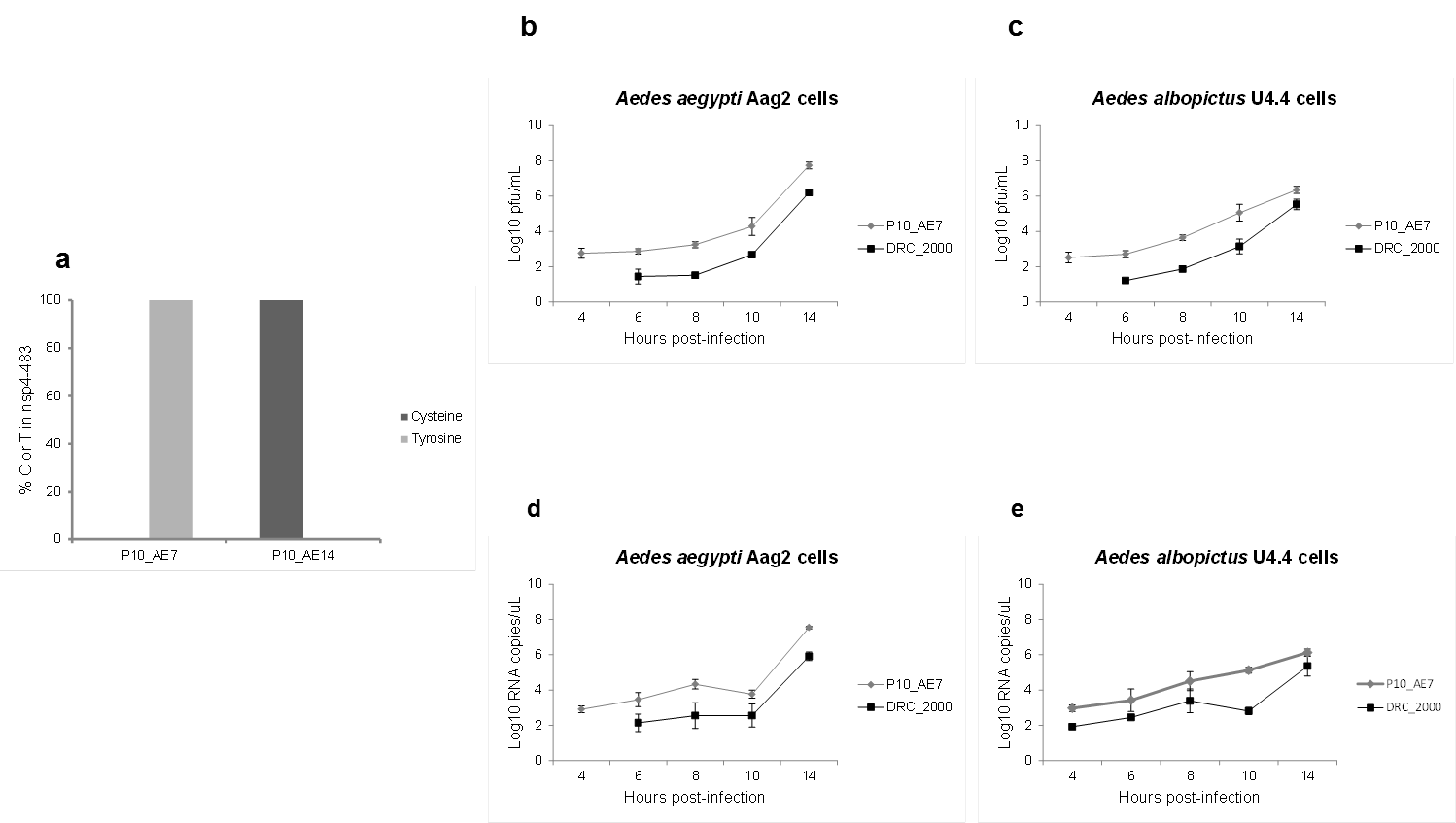


**Importance of mosquito quasispecies in selecting an epidemic arthropod-borne virus.**

Marie Vazeille^1^, Karima Zouache^1^, Anubis Vega-Rúa^1,2^ Jean-Michel Thiberge^3^, Valérie Caro^3^, André Yébakima^4^, Laurence Mousson^1^, Géraldine Piorkowski^5,6^, Catherine Dauga^7^, Marie-Christine Vaney^8^, Mosè Manni^9^, Giuliano Gasperi^9^, Xavier de Lamballerie^5,6^, and Anna-Bella Failloux^1,*^

**Supplementary Table 1 |Amino-acid substitutions in non-structural genes (a) and structural genes (b) of CHIKV strains, DRC_2000, P10_AE7, P10_AE14, P10_AL7, and P10_AL14 genomes**. Comparisons were performed with the S27 isolated in Tanzania in 1952.

(a)

| Strains |  |  | | Amino-acid position | | | | | | | | | | | | | | | | | | | | | | |
| --- | --- | --- | --- | --- | --- | --- | --- | --- | --- | --- | --- | --- | --- | --- | --- | --- | --- | --- | --- | --- | --- | --- | --- | --- | --- | --- |
|  |  | **nsp1** | | | | | | | | | | | |  | **nsp2** | | | | | | **nsp3** | | | | | |
|  | 6 | | 75 | | 114 | 172 | 234 | 301 | 314 | 383 | 384 | 481 | 507 | 57 | 78 | 374 | 510 | 642 | 643 | 685 | 117 | 175 | 326 | 328 | 331 | 337 |
| DRC_2000 | - | | D->E | | G->E | L->V | E->K | - | M->T | M->L | I->L | T->I | L->R | **-** | S->P | H->Y | - | C->Y | S->N | - | - | V->I | P->S | Q->P | V->A | T->A |
| P10_AE7 | - | | D->E | | G->E | L->V | E->K | - | M->T | M->L | I->L | T->I | L->R | **A->T** | S->P | H->Y | K->T | C->Y | S->N | - | - | V->I | P->S | Q->P | V->A | T->A |
| P10_AE14 | - | | D->E | | G->E | L->V | E->K | **T->I** | - | M->L | I->L | T->I | L->R | - | S->P | H->Y | K->T | C->Y | S->N | - | - | V->I | P->S | Q->P | V->A | T->A |
| P10_AL7 | - | | D->E | | G->E | L->V | E->K | - | M->T | M->L | I->L | T->I | L->R | - | S->P | H->Y | K->T | C->Y | S->N | - | - | V->I | P->S | Q->P | V->A | T->A |
| P10_AL14 | **V->E** | | D->E | | G->E | L->V | E->K | - | M->T | M->L | I->L | T->I | L->R | - | S->P | H->Y | - | C->Y | S->N | **N->D** | **G->R** | V->I | P->S | Q->P | V->A | T->A |

| Strains |  |  |  |  |  | | Amino-acid position | | | | | | | | | | |
| --- | --- | --- | --- | --- | --- | --- | --- | --- | --- | --- | --- | --- | --- | --- | --- | --- | --- |
|  |  |  |  |  | **nsp3** | | | | | |  | **nsp4** | | | | | |
|  | 378 | 382 | 352 | 376 | 399 | 439 | | 449 | 462 | 483 | 524 | 93 | 483 | 500 | 514 | 555 | 604 |
| DRC_2000 | T->M | A->T | K->E | I->T | V->I | Q->R | | M->T | S->N | N->S | R->opal | - | - | Q->L | I->T | V->I | V->I |
| P10_AE7 | T->M | A->T | K->E | I->T | V->I | Q->R | | M->T | S->N | N->S | R->opal | P->S | **C->Y** | Q->L | I->T | V->I | V->I |
| P10_AE14 | T->M | A->T | K->E | I->T | V->I | Q->R | | M->T | S->N | N->S | R->opal | - | **-** | Q->L | I->T | V->I | V->I |
| P10_AL7 | T->M | A->T | K->E | I->T | V->I | Q->R | | M->T | S->N | N->S | R->opal | P->S | **-** | Q->L | I->T | V->I | V->I |
| P10_AL14 | T->M | A->T | K->E | I->T | V->I | Q->R | | M->T | S->N | N->S | R->opal | - | **-** | Q->L | I->T | V->I | V->I |

(b)

| Strains |  |  | Amino-acid position | | | | | | | | | | | | | | | | | | |
| --- | --- | --- | --- | --- | --- | --- | --- | --- | --- | --- | --- | --- | --- | --- | --- | --- | --- | --- | --- | --- | --- |
|  | **C** | **E3** | | **E2** | | | | | | | | | | | | | | | | | |
|  | 63 | 23 | | 57 | 74 | 79 | 118 | 149 | 160 | 164 | 181 | 194 | 217 | 222 | 249 | 262 | 267 | 299 | 344 | 377 | 415 |
| DRC_2000 | K->R | I->T | | G->K | I->T | G->E | S->G | K->R | N->T | A->T | L->M | S->G | - | V->I | - | A->V | M->R | S->N | A->T | I->V | I->L |
| P10_AE7 | K->R | I->T | | G->K | I->T | G->E | S->G | K->R | N->T | A->T | L->M | S->G | - | V->I | - | A->V | M->R | S->N | A->T | I->V | I->L |
| P10_AE14 | K->R | I->T | | G->K | I->T | G->E | S->G | K->R | N->T | A->T | L->M | S->G | **I->V** | V->I | **G->R** | A->V | M->R | S->N | A->T | I->V | I->L |
| P10_AL7 | K->R | I->T | | G->K | I->T | G->E | S->G | K->R | N->T | A->T | L->M | S->G | - | V->I | - | A->V | M->R | S->N | A->T | I->V | I->L |
| P10_AL14 | K->R | I->T | | G->K | I->T | G->E | S->G | K->R | N->T | A->T | L->M | S->G | - | V->I | - | A->V | M->R | S->N | A->T | I->V | I->L |

| Strains | Amino-acid position | | | | | |
| --- | --- | --- | --- | --- | --- | --- |
|  | **6K** | | **E1** | | | |
|  | 54 | 60 | 37 | 175 | 269 | 322 |
| DRC_2000 | I->V | S->N | T->I | N->S | M->V | V->A |
| P10_AE7 | I->V | S->N | T->I | N->S | M->V | V->A |
| P10_AE14 | I->V | S->N | T->I | N->S | M->V | V->A |
| P10_AL7 | I->V | S->N | T->I | N->S | M->V | V->A |
| P10_AL14 | I->V | S->N | T->I | N->S | M->V | V->A |

In bold: substitution specific to one viral strain; -: no substitution; DRC_2000 isolated in 2000 in the Democratic Republic of Congo belongs to the ECSA phylogroup and the Central African lineage; P10_AE7, 10^th^ passage between HFF cells and *Ae. aegypti* initiated with saliva collected at 7 dpi; P10_AE14; 10^th^ passage between HFF cells and *Ae. aegypti* initiated with saliva collected at 14 dpi, P10_AL7, 10^th^ passage between HFF cells and *Ae. albopictus* initiated with saliva collected at 7 dpi; P10_AL14,10^th^ passage between HFF cells and *Ae. albopictus* initiated with saliva collected at 14 dpi.

**Importance of mosquito quasispecies in selecting an epidemic arthropod-borne virus.**

Marie Vazeille^1^, Karima Zouache^1^, Anubis Vega-Rúa^1,2^ Jean-Michel Thiberge^3^, Valérie Caro^3^, André Yébakima^4^, Laurence Mousson^1^, Géraldine Piorkowski^5,6^, Catherine Dauga^7^, Marie-Christine Vaney^8^, Mosè Manni^9^, Giuliano Gasperi^9^, Xavier de Lamballerie^5,6^, and Anna-Bella Failloux^1,*^

**Extended Data Table 2 | Allelic frequencies of 11 microsatellites differentiating the 16 *Aedes albopictus* populations.**

**Locus: C103**

-----------------------------------------

Pop Alleles Genes

-----------------------------------------

181 183 185 189

-----------------------------------------

MIA 1.000 0.000 0.000 0.000 58

JRB 0.467 0.000 0.050 0.483 60

MAN 0.550 0.000 0.433 0.017 60

TYS 0.567 0.000 0.250 0.183 60

PNA 0.817 0.000 0.100 0.083 60

VRB 0.667 0.000 0.183 0.150 60

SAN 0.883 0.000 0.033 0.083 60

PNM 0.850 0.000 0.000 0.150 60

STR 0.850 0.000 0.050 0.100 60

BL 0.450 0.000 0.383 0.167 60

CAL 0.433 0.000 0.550 0.017 60

CONG 0.367 0.000 0.633 0.000 60

MXA 0.433 0.017 0.550 0.000 60

ALPROV 0.433 0.000 0.567 0.000 60

MFILOU 0.300 0.000 0.633 0.067 60

STANDR 0.517 0.000 0.483 0.000 60

----------------------------------------

**Locus: C104**

-----------------------------------------------------------------------------------------------------------------------------------------

Pop Alleles Genes

-----------------------------------------------------------------------------------------------------------------------------------------

179 191 193 197 199 203 205 207 209 211 213 215 219 223 229 231 233 235 249 253

-----------------------------------------------------------------------------------------------------------------------------------------

MIA 0.000 0.000 0.000 0.397 0.017 0.362 0.034 0.000 0.190 0.000 0.000 0.000 0.000 0.000 0.000 0.000 0.000 0.000 0.000 0.000 58

JRB 0.000 0.000 0.000 0.000 0.050 0.533 0.200 0.017 0.117 0.000 0.067 0.017 0.000 0.000 0.000 0.000 0.000 0.000 0.000 0.000 60

MAN 0.000 0.000 0.000 0.067 0.000 0.367 0.233 0.083 0.183 0.000 0.017 0.000 0.000 0.000 0.033 0.000 0.000 0.017 0.000 0.000 60

TYS 0.000 0.000 0.033 0.000 0.000 0.167 0.200 0.000 0.433 0.000 0.167 0.000 0.000 0.000 0.000 0.000 0.000 0.000 0.000 0.000 60

PNA 0.000 0.000 0.000 0.000 0.000 0.467 0.000 0.000 0.533 0.000 0.000 0.000 0.000 0.000 0.000 0.000 0.000 0.000 0.000 0.000 60

VRB 0.000 0.000 0.000 0.000 0.083 0.117 0.083 0.017 0.517 0.000 0.183 0.000 0.000 0.000 0.000 0.000 0.000 0.000 0.000 0.000 60

SAN 0.000 0.000 0.000 0.000 0.000 0.533 0.117 0.000 0.283 0.000 0.067 0.000 0.000 0.000 0.000 0.000 0.000 0.000 0.000 0.000 60

PNM 0.000 0.000 0.000 0.000 0.000 0.250 0.233 0.000 0.267 0.000 0.133 0.000 0.117 0.000 0.000 0.000 0.000 0.000 0.000 0.000 60

STR 0.000 0.000 0.000 0.033 0.017 0.267 0.000 0.000 0.233 0.000 0.450 0.000 0.000 0.000 0.000 0.000 0.000 0.000 0.000 0.000 60

BL 0.000 0.017 0.050 0.000 0.017 0.083 0.117 0.000 0.433 0.000 0.017 0.017 0.067 0.133 0.000 0.000 0.033 0.000 0.017 0.000 60

CAL 0.000 0.000 0.000 0.067 0.050 0.200 0.683 0.000 0.000 0.000 0.000 0.000 0.000 0.000 0.000 0.000 0.000 0.000 0.000 0.000 60

CONG 0.000 0.000 0.000 0.017 0.033 0.417 0.200 0.033 0.000 0.000 0.000 0.000 0.000 0.000 0.083 0.017 0.000 0.133 0.000 0.067 60

MXA 0.000 0.000 0.000 0.000 0.000 0.083 0.217 0.033 0.667 0.000 0.000 0.000 0.000 0.000 0.000 0.000 0.000 0.000 0.000 0.000 60

ALPROV 0.000 0.000 0.000 0.183 0.017 0.067 0.083 0.100 0.167 0.017 0.050 0.000 0.067 0.167 0.000 0.083 0.000 0.000 0.000 0.000 60

MFILOU 0.000 0.000 0.000 0.050 0.000 0.633 0.017 0.250 0.050 0.000 0.000 0.000 0.000 0.000 0.000 0.000 0.000 0.000 0.000 0.000 60

STANDRE 0.017 0.000 0.000 0.167 0.017 0.083 0.017 0.000 0.283 0.017 0.000 0.017 0.283 0.017 0.000 0.083 0.000 0.000 0.000 0.000 60

-----------------------------------------------------------------------------------------------------------------------------------------

**Locus: C109**

-----------------------------------------------------------------------------------------------------------------------------------------------

Pop Alleles Genes

-------------------------------------------------------------------------------------------------------------------------------------

133 139 141 163 179 183 185 187 189 191 193 195 197 199 201 205 209 211 219 231 235

-----------------------------------------------------------------------------------------------------------------------------------------------

MIA 0.000 0.000 0.000 0.000 0.000 0.000 0.000 0.000 0.172 0.517 0.310 0.000 0.000 0.000 0.000 0.000 0.000 0.000 0.000 0.000 0.000 58

JRB 0.000 0.000 0.000 0.000 0.000 0.000 0.217 0.000 0.000 0.117 0.633 0.033 0.000 0.000 0.000 0.000 0.000 0.000 0.000 0.000 0.000 60

MAN 0.000 0.000 0.050 0.000 0.000 0.000 0.067 0.017 0.017 0.233 0.500 0.000 0.017 0.000 0.067 0.000 0.033 0.000 0.000 0.000 0.000 60

TYS 0.000 0.000 0.583 0.000 0.000 0.000 0.000 0.000 0.000 0.000 0.000 0.283 0.000 0.000 0.000 0.117 0.000 0.000 0.000 0.017 0.000 60

PNA 0.000 0.000 0.167 0.000 0.000 0.000 0.000 0.000 0.000 0.000 0.000 0.467 0.000 0.000 0.000 0.133 0.233 0.000 0.000 0.000 0.000 60

VRB 0.000 0.033 0.133 0.000 0.000 0.000 0.000 0.000 0.067 0.117 0.333 0.150 0.000 0.000 0.017 0.000 0.117 0.000 0.000 0.033 0.000 60

SAN 0.000 0.000 0.000 0.000 0.000 0.000 0.017 0.000 0.200 0.133 0.450 0.050 0.000 0.000 0.000 0.000 0.000 0.000 0.000 0.150 0.000 60

PNM 0.000 0.000 0.000 0.000 0.000 0.000 0.100 0.000 0.000 0.217 0.350 0.033 0.000 0.000 0.000 0.017 0.283 0.000 0.000 0.000 0.000 60

STR 0.000 0.000 0.033 0.000 0.000 0.000 0.000 0.000 0.133 0.450 0.317 0.017 0.000 0.000 0.000 0.017 0.000 0.000 0.000 0.033 0.000 60

BL 0.133 0.000 0.033 0.000 0.000 0.017 0.000 0.000 0.317 0.083 0.283 0.100 0.033 0.000 0.000 0.000 0.000 0.000 0.000 0.000 0.000 60

CAL 0.000 0.000 0.617 0.000 0.000 0.000 0.000 0.000 0.000 0.217 0.133 0.033 0.000 0.000 0.000 0.000 0.000 0.000 0.000 0.000 0.000 60

CONG 0.000 0.000 0.050 0.000 0.000 0.000 0.117 0.000 0.000 0.033 0.717 0.000 0.000 0.000 0.033 0.000 0.050 0.000 0.000 0.000 0.000 60

MXA 0.000 0.000 0.000 0.017 0.100 0.000 0.000 0.000 0.050 0.133 0.017 0.067 0.000 0.017 0.017 0.017 0.383 0.017 0.017 0.150 0.000 60

ALPROV 0.000 0.000 0.000 0.000 0.000 0.000 0.034 0.000 0.017 0.345 0.190 0.052 0.259 0.000 0.103 0.000 0.000 0.000 0.000 0.000 0.000 58

MFILOUF 0.000 0.000 0.200 0.000 0.000 0.000 0.183 0.100 0.000 0.267 0.033 0.100 0.000 0.000 0.117 0.000 0.000 0.000 0.000 0.000 0.000 60

STANDRE 0.000 0.000 0.117 0.000 0.000 0.000 0.000 0.000 0.000 0.433 0.100 0.033 0.117 0.033 0.050 0.000 0.000 0.000 0.000 0.017 0.100 60

-----------------------------------------------------------------------------------------------------------------------------------------------

**Locus: C4**

-----------------------------------------------------------

Pop Alleles Genes

-----------------------------------------------------------

104 132 134 136 138 140 142

MIA 0.000 0.000 0.000 1.000 0.000 0.000 0.000 58

JRB 0.000 0.000 0.000 1.000 0.000 0.000 0.000 60

MAN 0.000 0.000 0.000 1.000 0.000 0.000 0.000 60

TYS 0.000 0.000 0.233 0.733 0.033 0.000 0.000 60

PNA 0.000 0.000 0.133 0.867 0.000 0.000 0.000 60

VRB 0.000 0.000 0.033 0.867 0.100 0.000 0.000 60

SAN 0.000 0.000 0.000 1.000 0.000 0.000 0.000 60

PNM 0.000 0.033 0.000 0.967 0.000 0.000 0.000 60

STR 0.000 0.000 0.000 1.000 0.000 0.000 0.000 60

BL 0.000 0.000 0.000 1.000 0.000 0.000 0.000 60

CAL 0.000 0.000 0.000 1.000 0.000 0.000 0.000 60

CONG 0.000 0.000 0.000 1.000 0.000 0.000 0.000 60

MXA 0.033 0.183 0.150 0.500 0.000 0.083 0.050 60

ALPROV 0.000 0.033 0.000 0.967 0.000 0.000 0.000 60

MFILOUF 0.000 0.000 0.000 1.000 0.000 0.000 0.000 60

STANDRE 0.000 0.000 0.000 1.000 0.000 0.000 0.000 60

------------------------------------------------------------

**Locus: D4**

-----------------------------------------------------------------------------------

Pop Alleles Genes

----------------------------------------------------------------------

196 198 202 204 206 208 210 212 220 222 224

---------------------------------------------------------------------------------

MIA 0.000 0.000 0.000 0.000 0.207 0.017 0.776 0.000 0.000 0.000 0.000 58

JRB 0.000 0.000 0.000 0.200 0.233 0.000 0.567 0.000 0.000 0.000 0.000 60

MAN 0.000 0.000 0.000 0.200 0.150 0.367 0.233 0.017 0.000 0.033 0.000 60

TYS 0.000 0.000 0.000 0.200 0.083 0.167 0.133 0.000 0.000 0.417 0.000 60

PNA 0.000 0.000 0.000 0.000 0.200 0.800 0.000 0.000 0.000 0.000 0.000 60

VRB 0.000 0.000 0.000 0.300 0.217 0.283 0.200 0.000 0.000 0.000 0.000 60

SAN 0.000 0.000 0.000 0.117 0.117 0.000 0.417 0.000 0.000 0.000 0.350 60

PNM 0.000 0.000 0.000 0.500 0.050 0.200 0.250 0.000 0.000 0.000 0.000 60

STR 0.000 0.000 0.000 0.000 0.283 0.000 0.717 0.000 0.000 0.000 0.000 60

BL 0.000 0.000 0.000 0.233 0.317 0.133 0.117 0.000 0.067 0.133 0.000 60

CAL 0.000 0.000 0.000 0.367 0.100 0.017 0.183 0.200 0.133 0.000 0.000 60

CONG 0.000 0.000 0.000 0.233 0.000 0.533 0.017 0.117 0.000 0.100 0.000 60

MXA 0.017 0.017 0.000 0.600 0.050 0.300 0.017 0.000 0.000 0.000 0.000 60

ALPROV 0.000 0.000 0.000 0.086 0.121 0.207 0.345 0.000 0.052 0.172 0.017 58

MFILOUF 0.000 0.000 0.000 0.017 0.117 0.667 0.150 0.033 0.000 0.017 0.000 60

STANDRE 0.000 0.000 0.033 0.033 0.133 0.183 0.233 0.000 0.050 0.183 0.150 60

---------------------------------------------------------------------------------

**Locus: C115**

-----------------------------------------------------------------------------

Pop Alleles Genes

-----------------------------------------------------------------------------

239 241 243 245 247 249 253 257 261 265

-----------------------------------------------------------------------------

MIA 0.000 0.000 0.000 0.000 0.000 0.000 0.000 0.862 0.000 0.138 58

JRB 0.000 0.000 0.033 0.000 0.017 0.000 0.000 0.550 0.100 0.300 60

MAN 0.000 0.000 0.017 0.000 0.000 0.000 0.100 0.583 0.167 0.133 60

TYS 0.000 0.000 0.000 0.000 0.000 0.000 0.133 0.867 0.000 0.000 60

PNA 0.000 0.000 0.083 0.000 0.000 0.000 0.150 0.433 0.333 0.000 60

VRB 0.017 0.017 0.117 0.033 0.000 0.000 0.067 0.583 0.083 0.083 60

SAN 0.000 0.000 0.183 0.000 0.067 0.000 0.000 0.050 0.117 0.583 60

PNM 0.000 0.000 0.000 0.017 0.167 0.000 0.000 0.450 0.367 0.000 60

STR 0.000 0.000 0.000 0.133 0.017 0.000 0.000 0.317 0.250 0.283 60

BLM 0.000 0.000 0.000 0.017 0.000 0.017 0.067 0.783 0.050 0.067 60

CAL 0.000 0.000 0.000 0.000 0.000 0.000 0.000 0.417 0.200 0.383 60

CONG 0.000 0.000 0.000 0.000 0.000 0.000 0.250 0.600 0.150 0.000 60

MXA 0.000 0.000 0.033 0.000 0.000 0.000 0.183 0.383 0.400 0.000 60

ALPROV 0.000 0.000 0.000 0.000 0.000 0.000 0.056 0.537 0.167 0.241 54

MFILOUF 0.000 0.000 0.033 0.000 0.000 0.000 0.217 0.650 0.033 0.067 60

STANDRE 0.000 0.000 0.000 0.000 0.000 0.000 0.017 0.750 0.117 0.117 60

-----------------------------------------------------------------------------

**Locus: B3**

----------------------------------------------------------------------------------

Pop Alleles Genes

----------------------------------------------------------------------------------

141 143 147 149 151 153 155 157 159 161 163

----------------------------------------------------------------------------------

MIA 0.000 0.000 0.000 0.000 0.724 0.276 0.000 0.000 0.000 0.000 0.000 58

JRB 0.000 0.000 0.000 0.000 0.700 0.300 0.000 0.000 0.000 0.000 0.000 60

MAN 0.000 0.000 0.000 0.000 0.867 0.133 0.000 0.000 0.000 0.000 0.000 60

TYS 0.000 0.000 0.000 0.000 0.967 0.017 0.000 0.000 0.000 0.017 0.000 60

PNA 0.000 0.000 0.000 0.000 0.850 0.133 0.017 0.000 0.000 0.000 0.000 60

VRB 0.117 0.000 0.017 0.000 0.817 0.017 0.000 0.033 0.000 0.000 0.000 60

SAN 0.000 0.000 0.000 0.000 0.567 0.433 0.000 0.000 0.000 0.000 0.000 60

PNM 0.000 0.000 0.000 0.000 0.500 0.400 0.000 0.100 0.000 0.000 0.000 60

STR 0.000 0.000 0.000 0.000 0.100 0.900 0.000 0.000 0.000 0.000 0.000 60

BL 0.000 0.000 0.000 0.000 0.250 0.483 0.000 0.117 0.067 0.017 0.067 60

CAL 0.000 0.000 0.000 0.000 0.967 0.033 0.000 0.000 0.000 0.000 0.000 60

CONG 0.000 0.000 0.000 0.000 0.933 0.067 0.000 0.000 0.000 0.000 0.000 60

MXA 0.000 0.000 0.000 0.000 1.000 0.000 0.000 0.000 0.000 0.000 0.000 60

ALPROV 0.000 0.000 0.000 0.000 0.815 0.111 0.019 0.019 0.037 0.000 0.000 54

MFILOUF 0.000 0.017 0.017 0.000 0.550 0.383 0.033 0.000 0.000 0.000 0.000 60

STANDRE 0.017 0.000 0.000 0.133 0.583 0.150 0.033 0.083 0.000 0.000 0.000 60

----------------------------------------------------------------------------------

**Locus: C123**

-----------------------------------------------------------------------------

Pop Alleles Genes

-----------------------------------------------------------------------------

121 123 125 127 129 131 133 135 137 139

MIA 0.000 0.000 0.000 0.776 0.000 0.000 0.000 0.000 0.155 0.069 58

JRB 0.000 0.033 0.000 0.817 0.000 0.000 0.017 0.000 0.067 0.067 60

MAN 0.000 0.050 0.050 0.333 0.067 0.000 0.350 0.017 0.133 0.000 60

TYS 0.000 0.000 0.000 0.500 0.000 0.000 0.500 0.000 0.000 0.000 60

PNA 0.000 0.000 0.000 0.550 0.000 0.000 0.450 0.000 0.000 0.000 60

VRB 0.000 0.000 0.050 0.550 0.000 0.033 0.367 0.000 0.000 0.000 60

SAN 0.000 0.000 0.000 0.667 0.000 0.000 0.200 0.000 0.017 0.117 60

PNM 0.000 0.050 0.167 0.567 0.000 0.000 0.117 0.000 0.050 0.050 60

STR 0.000 0.000 0.000 0.450 0.000 0.000 0.000 0.000 0.000 0.550 60

BL 0.017 0.000 0.067 0.400 0.017 0.017 0.400 0.083 0.000 0.000 60

CAL 0.150 0.050 0.000 0.583 0.017 0.150 0.050 0.000 0.000 0.000 60

CONG 0.000 0.033 0.033 0.183 0.133 0.133 0.183 0.033 0.033 0.233 60

MXA 0.000 0.000 0.017 0.583 0.000 0.000 0.400 0.000 0.000 0.000 60

ALPROV 0.000 0.000 0.103 0.448 0.034 0.190 0.224 0.000 0.000 0.000 58

MFILOUF 0.000 0.000 0.172 0.276 0.069 0.103 0.121 0.017 0.000 0.241 58

STANDRE 0.000 0.000 0.050 0.300 0.033 0.250 0.367 0.000 0.000 0.000 60

-----------------------------------------------------------------------------

**Locus: C112**

----------------------------------------------

Pop Alleles Genes

----------------------------------------------

223 227 231 233 235

-----------------------------------------------

MIA 0.000 0.000 0.000 1.000 0.000 58

JRB 0.000 0.000 0.000 1.000 0.000 60

MAN 0.000 0.000 0.000 1.000 0.000 60

TYS 0.000 0.000 0.000 1.000 0.000 60

PNA 0.000 0.000 0.000 1.000 0.000 60

VRB 0.000 0.000 0.000 1.000 0.000 60

SAN 0.017 0.017 0.000 0.967 0.000 60

PNM 0.000 0.000 0.000 0.950 0.050 60

STR 0.000 0.000 0.000 1.000 0.000 60

BL 0.000 0.000 0.000 1.000 0.000 60

CAL 0.000 0.000 0.017 0.983 0.000 60

CONG 0.000 0.000 0.000 1.000 0.000 60

MXA 0.000 0.000 0.000 1.000 0.000 60

ALPROV 0.000 0.000 0.000 1.000 0.000 58

MFILOUF 0.000 0.000 0.000 0.983 0.017 60

STANDRE 0.000 0.017 0.000 0.983 0.000 60

-----------------------------------------------

**Locus: D8**

-----------------------------------------------------------------------------------------------------------------------------------------------

Pop Alleles Genes

-----------------------------------------------------------------------------------------------------------------------------------------------

210 216 220 224 226 228 230 232 234 238 240 242 244 246 248 250 252 254 260 266 268

MIA 0.000 0.000 0.000 0.000 0.000 0.000 0.000 0.000 0.000 0.000 0.000 0.310 0.000 0.000 0.000 0.483 0.000 0.000 0.000 0.052 0.155 58

JRB 0.000 0.000 0.000 0.000 0.000 0.317 0.000 0.000 0.000 0.233 0.000 0.350 0.000 0.000 0.000 0.033 0.000 0.000 0.067 0.000 0.000 60

MAN 0.000 0.000 0.000 0.000 0.000 0.317 0.000 0.033 0.000 0.100 0.000 0.350 0.000 0.033 0.000 0.100 0.000 0.000 0.000 0.000 0.067 60

TYS 0.050 0.000 0.000 0.017 0.017 0.250 0.083 0.000 0.000 0.067 0.117 0.117 0.083 0.200 0.000 0.000 0.000 0.000 0.000 0.000 0.000 60

PNA 0.000 0.000 0.000 0.000 0.000 0.000 0.000 0.000 0.000 0.000 0.000 0.433 0.467 0.100 0.000 0.000 0.000 0.000 0.000 0.000 0.000 60

VRB 0.000 0.000 0.000 0.000 0.000 0.350 0.017 0.000 0.000 0.117 0.000 0.283 0.133 0.067 0.017 0.000 0.017 0.000 0.000 0.000 0.000 60

SAN 0.000 0.000 0.000 0.000 0.000 0.067 0.000 0.000 0.000 0.117 0.000 0.683 0.000 0.000 0.000 0.083 0.000 0.050 0.000 0.000 0.000 60

PNM 0.000 0.000 0.000 0.000 0.000 0.100 0.000 0.000 0.000 0.000 0.000 0.850 0.000 0.050 0.000 0.000 0.000 0.000 0.000 0.000 0.000 60

STR 0.000 0.000 0.000 0.000 0.000 0.017 0.000 0.000 0.000 0.067 0.000 0.850 0.000 0.000 0.000 0.000 0.000 0.000 0.000 0.000 0.067 60

BL 0.017 0.000 0.000 0.000 0.000 0.033 0.000 0.000 0.000 0.217 0.000 0.367 0.000 0.150 0.000 0.000 0.217 0.000 0.000 0.000 0.000 60

CAL 0.017 0.067 0.000 0.000 0.000 0.000 0.000 0.000 0.000 0.317 0.000 0.283 0.000 0.050 0.000 0.267 0.000 0.000 0.000 0.000 0.000 60

CONG 0.000 0.000 0.033 0.000 0.000 0.417 0.000 0.100 0.050 0.050 0.000 0.217 0.000 0.017 0.000 0.067 0.050 0.000 0.000 0.000 0.000 60

MXA 0.000 0.000 0.000 0.000 0.000 0.100 0.000 0.000 0.000 0.450 0.000 0.200 0.000 0.117 0.000 0.000 0.000 0.133 0.000 0.000 0.000 60

ALPROV 0.050 0.000 0.000 0.000 0.000 0.050 0.000 0.000 0.000 0.067 0.000 0.450 0.000 0.033 0.317 0.000 0.000 0.000 0.000 0.000 0.033 60

MFILOUF 0.000 0.000 0.317 0.000 0.000 0.183 0.000 0.200 0.000 0.017 0.000 0.167 0.000 0.050 0.000 0.067 0.000 0.000 0.000 0.000 0.000 60

STANDRE 0.000 0.000 0.000 0.000 0.000 0.000 0.000 0.000 0.000 0.233 0.000 0.650 0.000 0.033 0.033 0.050 0.000 0.000 0.000 0.000 0.000 60

-----------------------------------------------------------------------------------------------------------------------------------------------

**Locus: B117**

-----------------------------------------------------

Pop Alleles Genes

-----------------------------------------------------

102 106 108 110 114 116

MIA 0.121 0.655 0.224 0.000 0.000 0.000 58

JRB 0.083 0.600 0.100 0.000 0.000 0.217 60

MAN 0.000 0.983 0.000 0.017 0.000 0.000 60

TYS 0.000 0.583 0.417 0.000 0.000 0.000 60

PNA 0.083 0.567 0.350 0.000 0.000 0.000 60

VRB 0.000 0.900 0.100 0.000 0.000 0.000 60

SAN 0.017 0.900 0.050 0.000 0.000 0.033 60

PNM 0.000 0.917 0.033 0.000 0.000 0.050 60

STR 0.000 0.783 0.183 0.000 0.000 0.033 60

BL 0.000 0.867 0.133 0.000 0.000 0.000 60

CAL 0.000 0.450 0.050 0.000 0.500 0.000 60

CONG 0.000 0.667 0.133 0.200 0.000 0.000 60

MXA 0.000 0.883 0.117 0.000 0.000 0.000 60

ALPROV 0.000 0.983 0.017 0.000 0.000 0.000 60

MFILOUF 0.000 0.900 0.100 0.000 0.000 0.000 60

STANDRE 0.000 0.833 0.167 0.000 0.000 0.000 60

-----------------------------------------------------

**Importance of mosquito quasispecies in selecting an epidemic arthropod-borne virus**

Marie Vazeille^1^, Karima Zouache^1^, Anubis Vega-Rúa^1,2^ Jean-Michel Thiberge^3^, Valérie Caro^3^, André Yébakima^4^, Laurence Mousson^1^, Géraldine Piorkowski^5,6^, Catherine Dauga^7^, Marie-Christine Vaney^8^, Mosè Manni^9^, Giuliano Gasperi^9^, Xavier de Lamballerie^5,6^, and Anna-Bella Failloux^1,*^

**Supplementary Table 3 | Genetic differences between the two CHIKV strains, DRC_2000 and Congo_2011.**

**Importance of mosquito quasispecies in selecting an epidemic arthropod-borne virus**

Marie Vazeille^1^, Karima Zouache^1^, Anubis Vega-Rúa^1,2^ Jean-Michel Thiberge^3^, Valérie Caro^3^, André Yébakima^4^, Laurence Mousson^1^, Géraldine Piorkowski^5,6^, Catherine Dauga^7^, Marie-Christine Vaney^8^, Mosè Manni^9^, Giuliano Gasperi^9^, Xavier de Lamballerie^5,6^, and Anna-Bella Failloux^1,*^

**Supplementary Table 4 |** Set of primers used for the sequencing of CHIKV strains.

| Sequence (5’ to 3’) | Forward/Reverse | nt position |
| --- | --- | --- |
| ATGGCTGCGTGAGACACAC | Forward 1 | 1-19 |
| TGAGACCACTGCCTATCATTTA | Reverse 1 | 3162 - 3183 |
| GACCTTCGATACATTCCAAA | Forward 2 | 3079 - 3098 |
| CACGTTGAATACTGCTGAGT | Reverse 2 | 6321- 6340 |
| ACACACTACAGAATGTACTGGCA | Forward 3 | 6243 - 6265 |
| CGTACATGAGTGACTAATCTTCCT | Reverse 3 | 8896 - 8919 |
| CAGCACCGTGTACGATTACTGG | Forward 4 | 8804 - 8825 |
| GTTCGAGAATCGTGGAAGAGTTC | Reverse 4 | 11874 - 11896 |
